# Unstable Gaze in Functional Dizziness: A Contribution to Understanding the Pathophysiology of Functional Disorders

**DOI:** 10.3389/fnins.2021.685590

**Published:** 2021-07-20

**Authors:** Lena Schröder, Dina von Werder, Cecilia Ramaioli, Thomas Wachtler, Peter Henningsen, Stefan Glasauer, Nadine Lehnen

**Affiliations:** ^1^Department of Psychosomatic Medicine and Psychotherapy, Klinikum Rechts der Isar, Technical University of Munich, Munich, Germany; ^2^Graduate School of Systemic Neurosciences, Ludwig-Maximilians-Universität München, Planegg-Martinsried, Germany; ^3^Department of Biology II, Ludwig-Maximilians-Universität München, Planegg-Martinsried, Germany; ^4^Institute of Medical Technology, Brandenburg University of Technology Cottbus-Senftenberg, Cottbus, Germany; ^5^Faculty of Health Sciences Brandenburg, Brandenburg University of Technology Cottbus-Senftenberg, Cottbus, Germany

**Keywords:** functional dizziness, pathophysiology, predictive coding, internal models, somatic symptom disorder, bodily distress disorder

## Abstract

**Objective:** We are still lacking a pathophysiological mechanism for functional disorders explaining the emergence and manifestation of characteristic, severely impairing bodily symptoms like chest pain or dizziness. A recent hypothesis based on the predictive coding theory of brain function suggests that in functional disorders, internal expectations do not match the actual sensory body states, leading to perceptual dysregulation and symptom perception. To test this hypothesis, we investigated the account of internal expectations and sensory input on gaze stabilization, a physiologically relevant parameter of gaze shifts, in functional dizziness.

**Methods:** We assessed gaze stabilization in eight functional dizziness patients and 11 healthy controls during two distinct epochs of large gaze shifts: during a counter-rotation epoch (CR epoch), where the brain can use internal models, motor planning, and resulting internal expectations to achieve internally driven gaze stabilization; and during an oscillation epoch (OSC epoch), where, due to terminated motor planning, no movement expectations are present, and gaze is stabilized by sensory input alone.

**Results:** Gaze stabilization differed between functional patients and healthy controls only when internal movement expectations were involved [*F*(1,17) = 14.63, *p* = 0.001, and partial η^2^ = 0.463]: functional dizziness patients showed reduced gaze stabilization during the CR (*p* = 0.036) but not OSC epoch (*p* = 0.26).

**Conclusion:** While sensory-driven gaze stabilization is intact, there are marked, well-measurable deficits in internally-driven gaze stabilization in functional dizziness pointing at internal expectations that do not match actual body states. This experimental evidence supports the perceptual dysregulation hypothesis of functional disorders and is an important step toward understanding the underlying pathophysiology.

## Introduction

A hallmark of functional disorders is the major discrepancy between patients’ very real suffering from bodily symptoms, like fatigue, bowel irritation, chest pain, or dizziness, and an unimpressive exam and clinical workup, which does not account for the symptoms. There is no clear pathophysiological correlate (Baizabal-Carvallo et al., 2019; Drane et al., 2020; Martin and Van Den Bergh, 2020) matching patients’ disability, distress, and lowered quality of life, which is often even more impaired than in patients with corresponding organic disorders (Carson et al., 2011; Vroegop et al., 2013). Diagnosis and, consequently, adequate treatment are typically delayed by many years. Such symptoms are common: dizziness, for example, has a lifetime prevalence of 30% (Neuhauser, 2009), and in 20–50% of the affected patients, symptoms are of functional nature (Staab and Ruckenstein, 2007; Stone et al., 2010). This comes with high psychiatric comorbidity (Eckhardt-Henn et al., 2003; Wiltink et al., 2009; Lahmann et al., 2015) and increased healthcare utilization (Wiltink et al., 2009). Traditionally, the absence of an explanatory organic impairment is part of the diagnostic criteria of functional disorders (e.g., in the current European diagnostic system ICD-10, World Health Organization, 2004). Today, we experience a major paradigm shift in clinical medicine, with positive signs becoming more and more important in the diagnosis of functional disorders (American Psychological Association, 2013; Stone, 2016; Stone et al., 2020). Within this paradigm shift, identifying a—potentially unifying—pathophysiological mechanism is of high clinical relevance, as it would help to improve the positive definition, swift diagnosis, and treatment of functional disorders.

**FIGURE 1:**
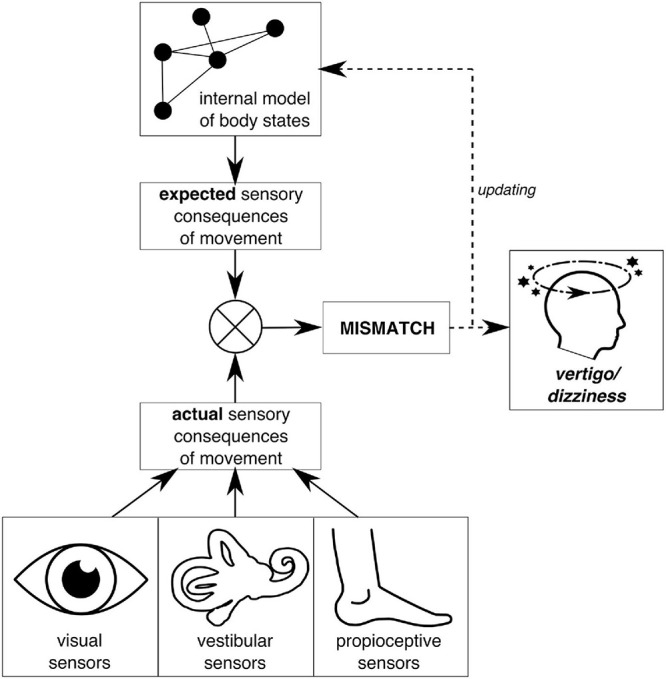
Schematic illustration of symptom emergence in the predictive coding framework on the example of vertigo/dizziness. Predictive coding understands perception as a constant interplay between incoming sensory information and internal expectations about such sensory input. In balance perception, for example, the actual sensory consequences of movement are processed by the visual, vestibular, and proprioceptive systems. Expectations about sensory consequences of movement are derived from internal models about the world and the body that constitute central nervous system (CNS)-internal representations of previously learned or experienced causal relations within the body, the environment, and their interaction. Ideally, such internal models match reality; i.e., they are a valid and reliable representation of the true causal relations. If this is not the case, resulting expectations about sensory input do not match the actual sensory activation. This mismatch, if not used as error signal to update internal models, can lead to persistent symptom experience, i.e., vertigo/dizziness.

A recent hypothesis reflecting this paradigm shift suggests that functional disorders emerge and manifest as a consequence of “perceptual dysregulation” in the central nervous system (CNS; Edwards et al., 2012; Van den Bergh et al., 2017; Henningsen et al., 2018; Pezzulo et al., 2019). Within the framework of predictive coding, central processing of incoming sensory information is biased by a mismatch resulting from incorrect internal expectations leading to symptom perception (Figure 1). Providing empirical validation of this hypothesis has been a current effort: several studies report “symptom-like” somatic illusions that could be evoked in healthy participants by experimentally altering internal expectations (e.g., Iodice et al., 2019; Bräscher et al., 2020; Wolters et al., 2020). Moreover, experimentally induced symptoms are more persistent in patients with functional disorders, uncoupled from corresponding sensory input (Bogaerts et al., 2010; Van Den Houte et al., 2018). The first evidence for altered sensorimotor processing is provided by our prior study investigating head control in patients with functional dizziness (Lehnen et al., 2019). When using combined eye–head movements to shift gaze to a new visual target, functional dizziness patients showed more pronounced head oscillations, a marker for the incongruency between sensory input and expectations in sensorimotor planning. This is a measurable marker clearly distinguishing functional patients from healthy controls. However, it does not identify the erroneous site within sensorimotor processing, which could be either faulty internal models or sensory input.

**FIGURE 2:**
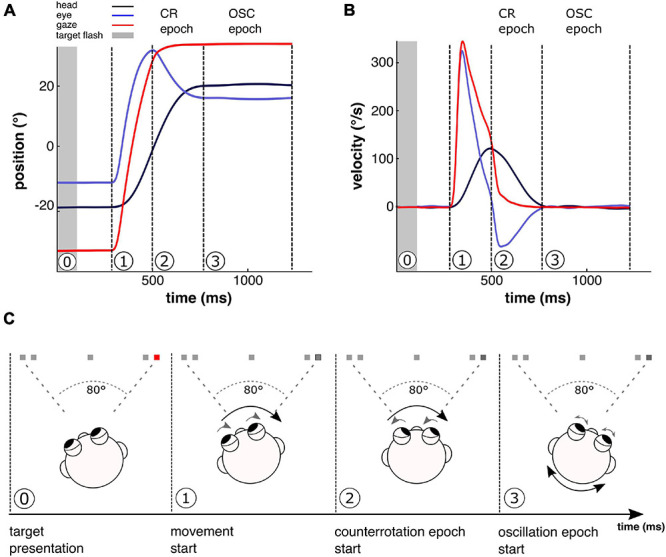
Movement sequence over the course of a single 80° gaze shift. Shown are position **(A)** and velocity traces **(B)** of experimentally recorded eye and head movements during one exemplary 80° gaze shift as well as computed gaze movement. Gaze, i.e., the position of the eyes in space, is composed of eye position (recorded in relation to the head) and head position (recorded in relation to space). An 80° gaze shift requires combined eye–head movements and follows a typical sequence **(C)**, including two distinct gaze stabilization epochs. Beginning from the target position of the previous trial, quickly after the flashed target light (**0**, gray bar in **A**,**B**, and red spot in **C**) is extinguished, eyes and head begin to move jointly toward the remembered target position (dark spot in **C**) in a coordinated and voluntarily planned way, representing the start of the gaze shift movement (**1**). Due to the active nature of head motion here, the vestibulo-ocular reflex (VOR) is suppressed (e.g., Angelaki and Cullen, 2008). When the gaze movement toward the target is finished, i.e., the eyes have reached maximum amplitude, but the head continues to move toward the target, the eyes counteract the continuing head movement by a counter-rotation (CR) in order to achieve stable gaze in this first stabilization epoch. Like the joint eye and head movement in epoch 1, the coordinated eye–head movements in this CR epoch are part of the active gaze shift, where movements are voluntarily planned, initiated, and executed to shift gaze toward the target position. Therefore, for gaze stabilization, motor planning is used to expect the sensory consequences of the head movement (e.g., Shanidze et al., 2010; King and Shanidze, 2011). The contribution of motor planning information on gaze stabilization in the CR epoch of this experimental paradigm has been demonstrated previously in bilateral vestibular loss patients (Saǧlam and Lehnen, 2014). Due to ongoing active head motion here, VOR is still suppressed in the CR epoch, although suppression is likely to be attenuated toward the end of the active movement (e.g., Lefèvre et al., 1992). When the head has finished its motion toward the target position, the active movement is completed (**3**). Now, the second stabilization epoch begins, where the eyes counteract small, unexpected passive head oscillations, further provoked by experimentally increased head inertia, which do not emerge as a consequence of motor planning of the active gaze shift. In this oscillation (OSC) epoch, in contrast to the CR epoch, no head movements are expected. Compensatory eye movements are driven by sensory feedback loops, mainly the VOR that is not suppressed anymore.

In the current paper, we assess a physiologically relevant parameter (gaze stability) in functional dizziness patients that helps to uncover this site. In our assessment, we make use of the fact that gaze stability in the context of an eye–head gaze shift to a new visual target is achieved in two epochs (Figure 2): first, a counter-rotation (CR) epoch, which is part of the planned movement toward the target, which means that efference copies and internal models can help to stabilize gaze (e.g., Roy and Cullen, 2004; Shanidze et al., 2010; King and Shanidze, 2011); second, an oscillation (OSC) epoch, where no self-initiated movements are expected, and stabilization thus depends on sensory feedback alone, i.e., mainly the vestibulo-ocular reflex.

Internal model and sensory input contribution to these two gaze stabilization epochs have been validated in a previous study using the same experimental design (Saǧlam and Lehnen, 2014): patients with complete bilateral vestibular loss show better gaze stabilization in the CR epoch than the OSC epoch, confirming the contribution of internal model and efference copy use in this stabilization epoch. Based on the “perceptual dysregulation” theory (Edwards et al., 2012; Van den Bergh et al., 2017; Henningsen et al., 2018; Pezzulo et al., 2019), during large eye–head gaze shifts, we expect functional dizziness patients to rely on incorrect internal models of their head, thus showing unstable gaze during the CR, but not the OSC epoch.

## Materials and Methods

This study investigates a dataset from patients with functional dizziness that has also been used in a prior publication (Lehnen et al., 2019). In this former publication, only head movement characteristics were analyzed. Now, we analyze further parameters from this dataset, as described in the following.

### Subjects

Eight patients with functional dizziness (aged 35 ± 13 years, mean ± SD, five females) that corresponded to the criteria for persistent postural-perceptual dizziness of the Bárány Society (Staab et al., 2017) and 11 age- and gender-matched healthy subjects (aged 32 ± 6 years, mean ± SD, six females) were included. Functional dizziness patients were recruited from the German Center for Vertigo and Balance Disorders, a tertiary vertigo/dizziness center of the University Hospital of Munich where they presented with permanent dizziness symptoms (>3 months). Only patients without any known prior or current structural peripheral or central vestibular dysfunction were included. History and an extensive clinical workup including neurological exams, neuro-ophthalmological and neuro-otological exams, caloric irrigation, subjective visual vertical, laser ophthalmoscopy, posturography, video head impulse test (vHIT), head impulse testing device—functional test (HITD-FT; after Ramaioli et al., 2014), and cranial magnetic resonance imaging (MRI) did not show any organ pathology. Healthy subjects, employees of the University Hospital of Munich who voluntarily participated in the study, reported no history of balance disorders and had a normal neurological exam. To ensure a structurally intact vestibular system on the day of examination, a vHIT was conducted prior to study conduction according to the EyeSeeCam vHIT manual (EyeSeeTec GmbH, Munich, Germany), revealing no deficits in functional dizziness patients [VOR gain at 0.06 s: left side: 1.02 ± 0.03, right side: 0.96 ± 0.04, mean, and standard error of the mean (SEM)] as well as healthy controls (VOR gain at 0.06 s: left side: 1.02 ± 0.02, right side: 0.98 ± 0.01).

All subjects gave their written consent prior to the study’s data collection. The study protocol was approved by the Ethics Committee of the University of Munich, the study design is in line with the Declaration of Helsinki.

### Experimental Procedure

Participants performed large horizontal (combined eye–head) gaze shifts toward visual targets, which were flashed in complete darkness (analogously to Lehnen, 2006). Subjects were seated in front of a desk at 1-m distance, with five light-emitting diodes (LEDs) placed at eye level in a line on the desk (one central and four peripheral LEDs, in 0.7- and 0.83-m distance left and right to the central LED), so that target eccentricity amounted to 0°, 35°, and 40° to the left and right with respect to participant’s middle head position. One experimental round consisted of 52 gaze shifts, with the target lights flashing consecutively in randomized order (amounting to gaze shifts of 35°, 40°, 70°, 75°, and 80° magnitude) and with randomized time interval between flashing lights (1.2–1.8 s) in order to prevent anticipation. Each target light was flashed for less than 0.1 s to avoid visual feedback. Subjects were instructed to direct their gaze toward the flashing LEDs naturally, by engaging eye and head movements, and to keep final gaze position until the next target flash occurred. Every subject performed two rounds of the experiment: one in the natural condition (*unweighted*) and one with experimentally altered head characteristics (*weighted*). For the latter condition, a helmet with eccentrically placed masses on both sides was firmly attached to the subjects’ heads, increasing the head moment of inertia 3.3-fold. All participants were unexperienced with respect to the experimental design and had never worn the helmet before. Eye and head movements were recorded with the EyeSeeCam measuring system (EyeSeeTec GmbH, Munich, Germany), by tracking movements of the left eye with video-oculography and head movements with 3D inertial sensors (resting state noise 0°–0.3°/s, SD 0.07°/s), placed in the middle of the forehead, both with a sampling rate of 220 Hz.

### Data Analysis

Data were analyzed offline using MATLAB (MathWorks, Natick, MA, United States). Head velocity in the horizontal plane was directly derived from the horizontal inertial sensor of the EyeSeeCam measuring system. Head position was computed as the integral of head velocity over time for each time point, normalized by initial head position, where participants were asked to fixate the central LED for 10 s. Eye position was calculated from pupil rotation vectors, also normalized by initial eye position. Eye velocity was computed as the derivative of eye position at each time point. Both eye and head position and velocity were filtered with a low-pass Gaussian filter (cutoff frequency 20 Hz). Gaze position and velocity were then computed by adding up eye and head position and velocity, respectively, so that gaze (eye in space) corresponded to the sum of eye (eye in head) and head (head in space). Continuous data streams were cut into single trials, beginning with the LED onset and ending 0.1 s after the next LED onset, so that each trial represented one gaze shift. Only gaze shifts in response to 75° and 80° jumps (43 target trials) and fulfilling the requirement of a large gaze shift (i.e., measured amplitude of >40° amplitude) were considered for the analysis. To remove saccades during CR and OSC epochs, saccades were detected automatically with a gaze peak velocity criterion of 30°/s and with saccade start and end being defined as the last minimum before and the next minimum after gaze velocity peaks, respectively. Saccade detection was then inspected visually and corrected manually, by adding undetected saccades (<1% for all subjects) as well as correcting the detected minima (<1% for all subjects). Eye and head velocities during a saccade window were removed from the analysis.

Gaze gains were defined as the amount of compensatory eye movement in respect to head movement and were calculated as the slope of the linear regression between eye and head velocity profiles using the MATLAB built-in function *robustfit* (analogously to Saǧlam and Lehnen, 2014). Gaze gains were computed for two gaze stabilization epochs: the internally-driven CR epoch as part of the planned gaze shift, using internal expectations and sensory information for stabilization, and the sensory-driven OSC epoch for sensory-dependent gaze stabilization after gaze shift end. CR epoch begins when the eye has reached maximum amplitude, but the head continues to move toward the target (Figure 2, picture 2). This was implemented by using the time window between the eye maximum eccentricity point and the point where head velocity reached 0°/s. OSC epoch begins when the active head movement has been terminated but the head continues to move passively, i.e., due to unexpected OSCs induced by increased head inertia (Figure 2, picture 3). We defined this epoch as the time window from the first zero crossing of head velocity until 0.1 s after the next LED flash. This was done to make sure that we harvest the data as long as possible. For both epochs, the resulting gain displays the amount of compensatory eye movement in relation to the head movement, with zero reflecting no compensatory eye movement at all and one reflecting perfect compensation. Only gaze shifts where the point of eye maximum eccentricity as well as the first head zero crossing could be detected were considered for the analysis. Of 43 gaze shifts in total, 34 ± 2 (mean ± SEM) and 33 ± 2 trials were taken into the analysis of mean CR and OSC gains, respectively, with no significant group differences [Wilks’ lambda (1,17) = 0.79, *p* = 0.15].

### Statistical Analysis

The Shapiro–Wilk test was used for normality assessment in all factor groups. Differences in gaze gains for CR epoch and OSC epoch (within-factor *epoch*), unweighted and weighted condition (within-factor *weight*), and gaze shifts to the left and right side (within-factor *side*) were analyzed with a 2 × 2 × 2 repeated-measures ANOVA (rmANOVA). Group differences were analyzed by adding a between-subject factor (*group*: healthy subjects and patients with functional dizziness) to the rmANOVA. After a significant effect, for *post hoc* testing, Bonferroni-corrected comparisons were computed for the respective conditions. Significance levels were the same for each statistical test (*p* = 0.05).

Note that there are differences in gaze gains from the left and right side [main effect *side*: *F*(1,17) = 43.4, *p* < 0.001, and partial η^2^ = 0.72], which are known from vHIT testing (Park et al., 2019) and attributed to the asymmetric camera position in the EyeSeeCam system. Although there was a significant interaction of gaze shift side with group in the rmANOVA [*side* ^∗^ *group* interaction: *F*(1,17) = 9.96, *p* = 0.006, and partial η^2^ = 0.37], in *post hoc* testing, those group differences did not reach statistical significance for neither the left (*p* = 0.055) nor the right side (*p* = 0.44). We therefore consider gaze gain alterations to the left and right side as similar for all conditions, so that factor and group comparisons should not be affected. For better readability, gaze gains in the written text are reported for gaze shifts to the left side only.

## Results

**FIGURE 3:**
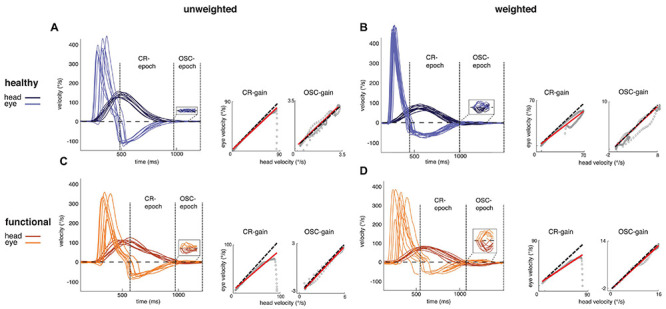
Filtered raw data of experimental movement recordings with illustrated gain computation. **(A–D** left) Shown are representative eye (light) and head (dark) velocity traces of one typical healthy subject **(A,B)** and one typical functional patient **(C,D)** for the unweighted (natural, **A,C**) and weighted condition (increased head inertia, **B,D**). The dashed horizontal lines display the zero line. Head oscillations—and counteracting eye movements—are illustrated in the window with increased y-axis scale (note that the functional dizziness patient display more pronounced head oscillations than the healthy participant, even in the natural condition. Group analysis confirming these differences have been published in Lehnen et al., 2019). **(A–D** right) Shown is eye velocity plotted against head velocity (gray circles) for counter-rotation (CR) and oscillation (OSC) gain computation for one representative gaze shift. Gaze gains are displayed as the slope of the solid lines, which represent the linear regression of eye velocity in head depending on head velocity in space. Perfect gaze stabilization, i.e., a gaze gain of 1, is indicated by the dashed line. The healthy subject shows intact CR-gaze stabilization in the unweighted condition, which is reduced by increasing the head inertia in the weighted condition. The functional patient displays reduced CR-gaze stabilization in the unweighted condition, which is further reduced in the weighted condition. During OSC epoch, both the healthy subject and the functional patient show intact gaze stabilization.

To investigate gaze stabilization during combined eye–head gaze shifts, we computed the amount of compensatory eye movements for gaze stabilization during two distinct epochs that either involve motor planning and internal expectations (internally-driven CR epoch) or not (sensory-driven OSC epoch). Figure 3 shows representative eye and head movements during such gaze shifts for one healthy participant (upper panels) and one functional dizziness patient (lower panels) in the natural condition (left) and with increased head inertia (right). In the natural, unweighted condition, the healthy participant performed compensatory eye movements in the CR epoch that counteract head movements and stabilize gaze. Increasing the head inertia led to a decrease of compensatory eye movements in the healthy subject. In the functional dizziness patient, compensatory eye movements in the CR epoch were already smaller in the natural, unweighted condition and further decreased with increased head inertia. In the OSC epoch, compensatory eye movements did not differ between the healthy subject and the functional dizziness patient.

**FIGURE 4:**
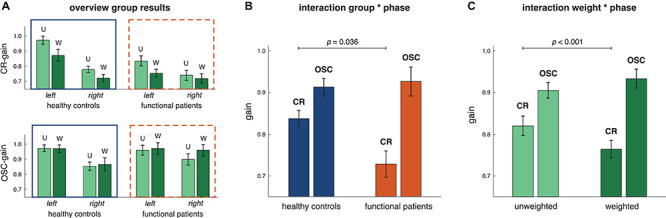
Results of group analysis (controls *n* = 11, patients *n* = 8). **(A)** Shown are gaze gains (mean and SEM) for all factor steps of the rmANOVA, i.e., gains to the left vs. right side (within-factor *side*, left group vs. right group of bars), unweighted (U) vs. weighted (W, within-factor *weight*, left vs. right bar within each bar group), in the CR vs. OSC epoch (within-factor *epoch*, upper vs. lower bar plot) for the healthy controls as well as the functional patients (between-factor *group*, all bars within solid vs. dashed squares). **(B)** Shown are gaze gains (mean and SEM) for the group * epoch interaction. Gaze gains differed between healthy controls and functional patients [*F*(1,17) = 14.63, *p* = 0.001, and partial η^2^ = 0.463]: functional patients displayed smaller gaze gains in the CR (*p* = 0.036) but not the OSC epoch (*p* = 0.26). **(C)** Shown are gaze gains (mean and SEM) for the weight * epoch interaction. Gaze gains differed between the unweighted and weighted conditions [*F*(1,17) = 20.24, *p* < 0.001; and partial η^2^ = 0.54], being reduced with weight in the CR (*p* < 0.001) but not the OSC epoch (*p* = 0.11).

These characteristics were found for all subjects (Figure 4). During CR epoch, healthy subjects showed a gain of 0.97 ± 0.03 (mean ± SEM) in the unweighted condition and 0.87 ± 0.04 in the weighted condition, and functional dizziness patients displayed a gain of 0.83 ± 0.04 in the unweighted and 0.75 ± 0.03 in the weighted condition. In contrast, during OSC epoch, gaze gains of healthy controls were 0.96 ± 0.02 in the unweighted and 0.97 ± 0.03 in the weighted condition and 0.95 ± 0.03 and 0.98 ± 0.04 in the unweighted and weighted condition of functional patients, respectively. RmANOVA confirmed different gaze gains for the CR and OSC epoch [main effect *epoch*: *F*(1,17) = 67.67, *p* < 0.001, and partial η^2^ = 0.80] influenced by group [*epoch* ^∗^ *group* interaction: *F*(1,17) = 14.63, *p* = 0.001, and partial η^2^ = 0.463]. *Post hoc* testing revealed that functional dizziness patients displayed significantly lower gaze stabilization than healthy subjects in the CR epoch (*p* = 0.036) but not the OSC epoch (*p* = 0.26). Increasing the head inertia influenced gaze stabilization in dependence of the epoch [*weight* ^∗^ *epoch* interaction: *F*(1,17) = 20.24, *p* < 0.001; and partial η^2^ = 0.54]. *Post hoc* tests showed reduced gaze stabilization with increased head inertia in the CR epoch (*p* < 0.001), but not in the OSC epoch (*p* = 0.11).

## Discussion

This study reveals marked deficits in gaze stabilization in functional dizziness patients. The deficits are only present during the internally-driven CR epoch of gaze shifts, where, based on motor planning and internal models, CNS expectations about the sensory outcome of the movement are used additionally to sensory input to stabilize gaze. During sensory-driven OSC epoch, when stabilization is only based on sensory input, gaze is stable.

As far as we know, this is the first study demonstrating a direct physiologically relevant pathology of functional dizziness. Importantly, this deficit is demonstrated in patients with a structurally fully intact peripheral and central vestibular system, as assessed by neurological, neuro-otological, and neuro-ophthalmological exams and an extensive workup, including subjective visual vertical, laser ophthalmoscopy, posturography, caloric irrigation, vHIT, HITD-FT, and cranial MRI. In analogy to the intact stabilization during the OSC epoch, vHIT, i.e., vestibular-driven ocular stabilization response to passive high-frequency head movements, was intact in these patients, also on the day of study.

Remarkably, however, during the CR epoch, where functional dizziness patients can use expectations together with sensory feedback for gaze stabilization, their deficits become visible and measurable: the eyes do not sufficiently counter-rotate to compensate for the head movement. As a consequence, gaze is not stable, but drifting. This effect—already present in the natural, unweighted condition—becomes even more pronounced when the head inertia is increased. In this weighted condition, when alterations in head characteristics are not yet reflected in CNS-internal representations, expectations are derived from the unweighted head internal model. Thus, wrong information is used to drive compensatory eye movements, leading to reduced gaze stabilization.

These findings demonstrate the significant role of both intact processing of vestibular feedback and expectation formation based on correct internal models, during eye–head gaze shifts. Their contribution over the course of the gaze shifts has been previously demonstrated within the same experimental paradigm, where patients with complete bilateral vestibular loss show gaze stabilization in the CR epoch despite missing sensory input (Saǧlam and Lehnen, 2014). Together with the present results, by using the example of functional dizziness patients, we are one step closer in locating an erroneous site of perceptual dysregulation in functional disorders (Edwards et al., 2012; Van den Bergh et al., 2017; Henningsen et al., 2018; Pezzulo et al., 2019). While we could provide evidence for a general central sensorimotor deficit in functional dizziness in a previous paper (Lehnen et al., 2019), we can now demonstrate first experimental evidence for an incorrect internal model use that has the potential to explain symptom experience in functional dizziness patients.

The idea of the role of mismatching information in symptom experience is central to the explanation of physiological and clinical vestibular vertigo. Vertigo is, by definition, a feeling of unsteadiness or movement, which occurs as a consequence of conflicting information in the CNS (Dieterich, 2004). Typically, by using expectations that rely on internal models about the body and the environment, the CNS establishes congruence between the different sensory or sensorimotor input sources, enabling stable positioning in and orientation within the environment. If the CNS fails to do so, e.g., in motion sickness (Money, 1970; Reason, 1978; Oman, 1982; Yardley, 1991; Oman and Cullen, 2014), the mismatch between expected and actual sensory input can elicit typical vertigo/dizziness feelings and nausea (Figure 1). Here, not only previous sensory experiences influence the expected sensory input but also higher-order cognitive motion beliefs, which are linked to certain contexts (Nooij et al., 2021). From this perspective, functional dizziness displays as a further dizziness/vertigo appearance, providing legitimation for the “realness” of symptom experience in patients with functional dizziness.

Studies investigating the direct pathophysiological mechanisms of functional dizziness are sparse. However, looking at imaging studies, several investigations report structural and functional brain alterations that can be related to our understanding of the underlying pathological mechanisms in functional dizziness patients. Structural gray matter decline (Wurthmann et al., 2017) as well as reduced functional resting state activity (Li et al., 2020) in functional dizziness patients were reported for brain areas that are important for spatial orientation and multisensory vestibular integration. Connectivity studies also demonstrated reduced resting-state functional connectivity between visual, vestibular, and spatial cognition areas (Lee et al., 2018; Li et al., 2020). Importantly, a special role of the cerebellum is highlighted (Lee et al., 2018; Huber et al., 2020): during a visual motion task, for example, cerebellar network activity of functional dizziness patients was reduced, whereas during static visual scenes, it was increased (Huber et al., 2020).

In our experiment, we were able to evoke unstable gaze in healthy controls, too: when head inertia was experimentally increased, our control subjects showed reduced compensatory eye movements in internally driven CR epoch and drifting gaze. The fact that creating a mismatch between expectations and actual sensory input by altering head mechanics is sufficient to reduce gaze stabilization provides further validation of our experimental paradigm as well as the supposed pathophysiological mechanism that underlies functional disorders. However, how this pathophysiological mechanism leads to symptom perception, remains to be seen. It is important to note that, while these findings have the potential to improve our understanding of “how” functional dizziness symptoms emerge and manifest, we cannot answer the “why” question of etiology. Furthermore, the interpretation of our study results presents only one possible explanation within a rather cognitive framework of symptom emergence and manifestation in patients with functional dizziness and does not exclude alternative interpretations. We understand this piece of evidence as a first experimental cornerstone that might guide future research toward transdiagnostic mechanisms for a positive definition of functional disorders. Further studies with functional dizziness patients as well as other patient groups are necessary to demonstrate the general validity of the perceptual dysregulation theory in functional disorders.

Nevertheless, we feel that an improved understanding of the pathophysiology of functional dizziness could constitute a great relief for both patients as well as caretakers. A measurable symptom correlate would most likely reduce stigma in this highly stigmatized patient group (Freidl et al., 2007; Rommelfanger et al., 2017; Eger Aydogmus, 2020). Also, providing measurable alterations has the potential of improving positive diagnosis of functional dizziness. In the long run, insights like these could further improve therapeutic strategies, e.g., in psychoeducation or sensorimotor adaptation training like it is already successfully done in unilateral and bilateral peripheral vestibular disorders (McDonnell and Hillier, 2007; Lehnen et al., 2018).

In summary, this study demonstrates unstable gaze in functional dizziness. During large eye-head gaze shifts toward visual targets gaze is unstable in the internally-driven CR epoch, i.e., when internal expectations are used to drive gaze stabilization, additionally to sensory input. In contrast, gaze is stable in the purely sensory-driven OSC epoch. Thereby, our findings provide further evidence for the predictive coding account of functional disorders, identifying—for the first time within the affected body system—internal expectations as the site where “perceptual dysregulation” arises (Edwards et al., 2012; Van den Bergh et al., 2017; Henningsen et al., 2018; Pezzulo et al., 2019). Together, these results have the potential to improve diagnosis and treatment in functional patients.

## Data Availability Statement

The original contributions presented in the study are publicly available. This data can be found here: https://doi.org/10.12751/g-node.sc1a64.

## Ethics Statement

This study involving human participants were reviewed and approved by Ethics Committee of the University of Munich. The patients/participants provided their written informed consent to participate in this study.

## Author Contributions

NL designed the study. CR collected the data. LS, DW, TW, SG, and NL analyzed the data. LS and DW created the figures. LS and NL wrote the initial manuscript. All authors reviewed and edited the manuscript.

## Conflict of Interest

NL and SG are shareholders of EyeSeeTec GmbH, manufacturers of the measurement system used. NL was a paid consultant and CR was a paid employee of EyeSeeTec GmbH. The remaining authors declare that the research was conducted in the absence of any commercial or financial relationships that could be construed as a potential conflict of interest.

## Abbreviations

CNS: central nervous system
CR: counter-rotation
HITD-FT: head impulse testing device—functional test
ICD-10: International Statistical Classification of Diseases and Health Related Problems 10
LED: light-emitting diode
MRI: magnetic resonance imaging
OSC: oscillation
rmANOVA: repeated-measures analysis of variance
SEM: standard error of the mean
vHIT: video head impulse Test
VOR: vestibulo-ocular reflex.
